# Expression of factors involved in apoptosis and cell survival is correlated with enzymes synthesizing lysophosphatidic acid and its receptors in granulosa cells originating from different types of bovine ovarian follicles

**DOI:** 10.1186/s12958-017-0287-9

**Published:** 2017-09-06

**Authors:** Emilia Sinderewicz, Katarzyna Grycmacher, Dorota Boruszewska, Ilona Kowalczyk-Zięba, Joanna Staszkiewicz, Tomasz Ślężak, Izabela Woclawek-Potocka

**Affiliations:** Department of Gamete and Embryo Biology, Institute of Animal Reproduction and Food Research, Polish Academy of Sciences, 10-747 Olsztyn, Poland

**Keywords:** Lysophosphatidic acid, Granulosa, Apoptosis, Proliferation, Ovarian follicle, Cow

## Abstract

**Background:**

Lysophosphatidic acid (LPA) regulates reproductive processes in the cow. Ovarian granulosa cells play a pivotal role in follicle growth and development. Nevertheless, the role of LPA in the local regulation of granulosa cell function in different follicle categories in the bovine ovary has not been investigated.

**Methods:**

Ovarian follicles were divided into healthy, transitional and atretic categories. The expression levels of AX, PLA2, LPARs and factors involved in apoptosis and cell survival processes in granulosa cells in different types of follicles were measured by real-time PCR. The correlations between the expression levels of AX, PLA2, LPARs and the examined factors were measured. The immunolocalization of AX, PLA2 and LPARs in different ovarian follicles was examined by immunohistochemistry. Statistical analyses were conducted in GraphPad using a one-way ANOVA followed by the Kruskal-Wallis multiple comparison test or a correlation analysis followed by Pearson’s test.

**Results:**

The expression levels of AX, PLA2 and LPARs, with the major role of LPAR2 and PLA2, were found in the granulosa cells originating from different follicle types. The expression levels of the factors involved in cell apoptosis (TNFα and its receptors, FAS, FASL, CASP3, CASP8, β-glycan, and DRAK2) were significantly higher in the granulosa cells of the atretic follicles compared to the healthy follicles. A number of correlations between LPARs, AX, PLA2 and factors associated with apoptosis were observed in the atretic but not in the healthy follicles. A greater expression of the factors involved in differentiation and proliferation in the granulosa cells (DICE1 and SOX2) was found in the healthy follicles in comparison with the atretic. A number of correlations between LPARs, AX, PLA2 and the factors associated with cell survival were observed in the healthy but not in the atretic follicles.

**Conclusions:**

Granulosa cells are the target of LPA action and the source of LPA synthesis in the bovine ovarian follicle. We suggest that the participation of LPA in apoptosis in the atretic follicles mainly occurs through the regulation of TNF-α-dependent and caspase-induced pathways. In the transitional follicles, LPA might influence the inhibins to shift the balance between the number of healthy and atretic follicles. In the healthy follicle type, LPA, acting via LPAR1, might regulate MCL1 and estradiol-stimulating ERβ mRNA expression, leading to the stimulation of anti-apoptotic processes in the granulosa cells and their differentiation and proliferation.

**Electronic supplementary material:**

The online version of this article (doi:10.1186/s12958-017-0287-9) contains supplementary material, which is available to authorized users.

## Background

The ovarian follicle is one of the most dynamically evolving structures in the female organism. This variability is possible because of the vital role of the proliferation, differentiation and apoptosis processes. The ovarian follicle is the functional unit of the ovary, developing during folliculogenesis. In mammals, ovarian folliculogenesis starts before or immediately after birth, when a number of small primordial follicles progress into large preovulatory follicles that enter the estrous cycle [1]. While primordial follicles are activated to grow into primary and secondary follicles, a single layer of flattened granulosa cells surrounding the oocyte, becomes cuboidal, proliferates and stratifies. After the formation of the secondary follicle, theca cells surround the granulosa cells and the antrum appears, allowing the follicle to ovulate [2]. Based on the estradiol:progesterone (E2:P4) ratio, ovarian follicles are divided into three groups, including healthy, transitional and atretic [3]. A high E2:P4 ratio is an indicator of healthy follicles and occurs in dominant follicles, which undergo ovulation. Therefore, we postulate that the category of the follicle reflects its quality, intensity of proliferation and apoptosis processes and, finally, its ability to release the oocyte, which can be successfully fertilized.

In cattle, follicles develop in a wave-like pattern [4]. Each follicle wave is characterized by the synchronous growth of a cohort of antral follicles, one of which is selected to become the dominant follicle, while all the other follicles regress and undergo apoptosis at various stages of the wave. Although atresia can emerge at any time during folliculogenesis, the majority of the follicles remain atretic throughout the antral stage [5].

One of the rapidly changing ovarian follicle components is the layer of granulosa cells, which are classified in the antral follicle as cumulus and mural granulosa. Granulosa cells are observed as the initial cell population undergoing apoptosis in atretic follicles before the oocyte and theca cells, which suggests their role as the initiator of follicular atresia [6–8]. On the other hand, in vivo and in vitro experiments show that various factors secreted from granulosa cells, including gonadal steroids, endocrine hormones and locally produced growth factors, are vital for the growth and survival of granulosa cells [9]. These widely studied hormones and growth factors are two gonadotrophins (luteinizing hormone (LH) and follicle stimulating hormone (FSH)); members of the transforming growth factor (TGF) superfamily (including activins, inhibins, and follistatin); fibroblast growth factor (FGF) family members; and the insulin-like growth factor (IGF) system, including the IGFs, their binding proteins and binding protein proteases [10, 11]. Follicle growth is also dependent on the steroidogenic capacity of the individual follicles [12] and the locally produced regulators that are derived from the theca layer, the granulosa cells, and the oocyte, playing a role in stimulating and/or inhibiting granulosa cell functions during ovarian follicular development [13, 14]. Our previous studies document that lysophosphatidic acid (LPA) is among the factors exerting auto/paracrine effect on the ovarian granulosa cells [15].

Lysophosphatidic acid is a small (molecular weight: 430–480 Da), bioactive glycerophospholipid built with one fatty acid chain and a phosphate group as a polar head [16, 17]; it is present in many eukaryotic tissues at low concentrations relative to major phospholipid species, and it occurs at higher concentrations (sub-micromolar range) in blood plasma [18, 19]. Lysophosphatidic acid is produced from the cellular membrane phospholipids by two major enzymes, namely autotaxin (AX) and phospholipase A (PLA) [20–22]. In mammals, LPA induces its action via the most well-known and featured high affinity G-protein-coupled receptor types, including LPAR1/EDG2, LPAR2/EDG4, LPAR3/EDG7 and LPAR4/P2Y9 [23–25]. Because of the heterogeneity of the receptor subtypes, expression patterns, and effector pathways, the effects of LPA are diverse and widespread, regulating many biological processes, including cell-to-cell interactions [26], tumorigenesis [27, 28] cell proliferation, differentiation [29, 30] and migration [31].

In the bovine reproductive tract, LPA is produced by the endometrium [25, 32–35], corpus luteum (CL) [36], ovary [15] and embryo [37]. In the bovine ovary, LPA stimulates E2 synthesis [15] in the granulosa cells of the bovine ovarian follicle and the MAPK ERK pathway via LPAR1 in the theca cells [38]. However, the potential influence of LPA on the granulosa cell functions, exerted from the specific follicle type, is still unknown.

Considering the major role of the granulosa cells in follicle development, the significance of LPA in the local regulation of the reproductive processes in cow and the potential importance of the follicle category in successful reproduction, the goal of this study was to investigate the expression of the enzymes responsible for LPA synthesis and its receptors in the granulosa cells originating from different bovine ovarian follicle types. We hypothesized that there was the follicle type-dependent expression of the enzymes producing LPA, the receptors for LPA and also the genes involved in cell proliferation and apoptosis in the granulosa cells. Furthermore, we supposed that there was a possible link between LPA synthesis and action and the expression of factors involved in cell survival and apoptosis.

## Methods

All of the experimental procedures were approved by the Local Animal Care and Use Committee in Olsztyn, Poland (Agreement No. 34/2012/N). Ovaries, irrespective of the stage of the estrous cycle, were collected from adult cows at a local abattoir and were transported to the laboratory in sterile NaCl at pH = 7.4 in 38 °C within 40 min.

### Follicular fluid, granulosa cells collection and follicle classification

The follicular fluid (FF) was aspirated from antral ovarian follicles (diameter < 5 mm) by syringe. The antral cavity of each follicle was flushed repeatedly with cold PBS to gain the granulosa cells, which were recovered by centrifugation of the follicular fluid at 1200 x G for 3 min and were pooled with the follicular fluid pellet.

The supernatant of the follicular fluid was collected and kept frozen at −20 °C until the concentrations of E2 and P4 were measured by the RIA method (using respectively: the DIAsource E2–RIA–CT Kit, KIP0629, Diasource and the DIAsource PROG–RIA–CT Kit, KIP1458, Diasource). Based on the intrafollicular E2:P4 ratio (according to [3]), the ovarian follicles were divided into three groups, including healthy, transitional and atretic (Table 2).

Granulosa cells obtained from the single follicles, categorized as healthy (*n* = 16), transitional (*n* = 16) or atretic (*n* = 16), were used for the study. The samples were collected in RNA later (R-0901, Sigma Aldrich) and were stored at −80 °C until the RNA extraction and gene transcription analysis by real-time RT-PCR.

### Real-time RT-PCR

Real-time RT-PCR was performed with an ABI Prism 7900 (Applied Biosystems, Life Technologies, USA) sequence detection system using the Maxima Probe/ROX qPCR Master Mix (#K0231, Thermo Scientific, USA). The PCR reactions were performed in 384-well plates. The results of the mRNA transcription were normalized to the β-actin (ACTB, an internal control) mRNA level and were expressed as arbitrary units. The housekeeping gene was chosen using the NormFinder software by comparing the following two candidate genes: GAPDH and β-actin [39]. The primers were designed using an online software package (http://frodo.wi.mit.edu/primer3/input.htm). The primer sequences and the sizes of the amplified fragments of all of the transcripts are shown in Table 1. For the relative quantification of mRNA abundance, Miner software was used (http://ewindup.info/miner/).

**Table 1:** Characteristics of real-time RT PCR

Gene symbol	GenBank accession No.	Forward primer/Reverse primer/Probe	Primer/probe size (bp)	Amplicon size (bp)
ACTB	AY141970.1	5’CGTCCACCGCAAATGCTT’3 5’TCTCGTTTTCTGCGCAAGTTAG’3 5’TAGGCGGACTGTTAGC’3	18 22 16	85
LPAR1	NM_174047.2	5’GTTCAACACAGGGCCCAATACT’3 5’AACCGTCAGGCTGGTGTCA’3 5’TGACTGTCAGCACGTGGCTCCTGC’3	22 19 24	85
LPAR2	NM_001192235.1	5’GTCTCTGAGCCTGCGGGTTA’3 5’TGACTTCAGAGCCCAATTCTTG’3 5’CATCTATGCAACTGGGAG’3	20 22 18	80
LPAR3	NM_001192741.2	5’TCAAGTAAAGCACGGGCAGAA’3 5’CGGGTGAGAACGCATTGTG’3 5’AGGACACGTGCATTTACAGA’3	21 19 20	90
LPAR4	NM_001098105.1	5’ATCACCAATCTGGCCCTCTCT’3 5’CAGTGGCGGTTGAAATTGTAAAA’3 5’CTCTTTGTCTGCACTCTA’3	21 23 18	80
AX	NM_001080293.1	5’CCATTATTCCCACAAGTTCACAGTA’3 5’TGTCAAGCATGTCACCAAAGGT’3 5’TGCCACAAAAGTGACAAA’3	19 20 18	80
PLA2	NM_001075864.1	5’CCATTATTCCCACAAGTTCACAGTA’3 5’TGTCAAGCATGTCACCAAAGGT’3 5’TGCCACAAAAGTGACAAA’3	25 22 18	80
TNFα	AC_000180.1	5’CCCTTCCACCCCCTTGTT’3 5’TGATGGCAGACAGGATGTTGA’3 5’CTCACCCACACCATCAGCCGCA’3	18 21 22	85
TNFα R1	U90937.1	5’AGAGACAGGACACCATCTGC’3 5’GCAAAGCCCGAAGACAATCA’3 5’ATGTCCAACCCGACCTTCAA’3	20 20 20	188
TNFα R2	NM_001040490.2	5’ATAGTGGGGAAAGGACAGGC’3 5’AGGAGTGGATTTGGAGGTGG’3 5’GGTACCCCAGTTAGCTCAGG’3	20 20 20	175
FAS	NM_174662.2	5’GCTAAGTCGCTTCAGTCGTG’3 5’GGATTGCTGAGTCGGACATG’3 5’CTGGAGTGGGTTGCCATTTC’3	20 20 20	179
FasL	NM_001098859.1	5’AGTACAAGAAGGGCAGCCTT’3 5’TCATCTTCCCCTCCATCAGC’3 5’GTCAGTCCTGCAACAACCAG’3	20 20 20	165
BCL2	U92434.1	5’TCTTTGAGTTCGGAGGGGTC’3 5’GGCCATACAGCTCCACAAAG’3 5’ATGACCGAGTACCTGAACCG’3	20 20 20	162
BAX	U92569.1	5’AACATGGAGCTGCAGAGGAT’3 5’CCAATGTCCAGCCCATGATG’3 5’AGTGGCGGCTGAAATGTTTT’3	20 20 20	208
CASP3	NM_001077840.1	5’TCTAAGCCATGGTGAAGAAGGAAT’3 5’GTCCCCTCTGAAGAAACTTGCTAA’3 5’TTTTGGAACCAACGGACCCGTCAA’3	24 24 24	84
CASP8	DQ319070.1	5’CTACTGAGAGAAGAGGCCCG’3 5’TTGATGGGTTCCTGCTTCCT’3 5’CTCAAGTTCCTAAGCCGGGA’3	20 20 20	210
B-glycan	NC_005113.4	5’TTCCAGGACGAACCCAACA’3 5’CCTCAACATAAACGTGTCCATTCT’3 5’CGTCACCTTCAACATGGAGCTGTACAACAC’3	19 24 30	86
DRAK2	AC_000159.1	5’TAACAGAGCAAATCGGACACAAG’3 5’ATTCCGCTTTGACGACTCGCTGCC’3 5’AGCAGCACGGTGTCCAAGA’3	22 24 19	79
MCL1	NM_001099206.1	5’CCAGGCTCGGACGGCTCGC’3 5’CTTTACCTTTGTTGGTCGGAGAA’3 5’TCTCCAGGGACTGCCGATAT’3	19 23 20	82
ERβ	NM_001001443.1	5’GCTGTCAGGCCTGTCGACTT’3 5’ATGCGGTACCCACACCTTTCT’3 5’CTATGAGGTTGGAATGGT’3	20 21 18	85
DICE1	AY386968.1	5’ACCGCCTTCACTGCCTTCT’3 5’AGATGAAAGATTTGCTGTCGATAGATT’3 5’CAGAGCTCGGACGGCTCGC’3	19 27 19	85
SOX2	AC_000158.1	5’CACCTGCAAGTTTCCTGCTT’3 5’TGTAAAATGTGTAGCCGCCG’3 5’CAGCATATTGACTCCACCGC’3	20 20 20	202

### Immunohistochemistry

To immunolocalize the enzymes involved in LPA production (AX and PLA_2_) and the LPA receptors (LPAR1–4) in the bovine ovarian follicle, the ovaries with antral follicles were fixed in buffered 4% formaldehyde for immunohistochemistry (according to [40]). The examined proteins were stained using the following antibodies: rabbit polyclonal: anti-LPAR1, anti-LPAR3 (dilution for two antibodies 1:50, Cayman, #10005280, #10004840, respectively), anti-LPAR2, anti-PLA2 (dilution for two antibodies 1:100, Santa Cruz Biotechnology, #sc-25,490, #sc-98,424, respectively), and anti-AX (dilution 1:50, Santa Cruz Biotechnology, #sc-50,366); goat polyclonal: anti-LPAR4 (dilution 1:100, Santa Cruz Biotechnology, #sc-46,021). The nNegative control sections were incubated with normal rabbit or goat irrelevant IgG (concentration 1:100; Santa Cruz Biotechnology; #sc-2027; #sc-2028, respectively).

### Statistical analyses

**Fig. 1:**
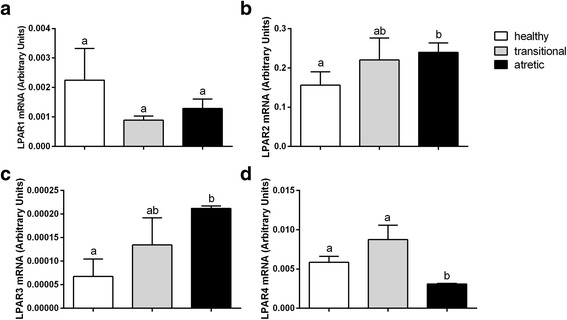
The mRNA abundance of the receptors for LPA, including LPAR1 (**a**), LPAR2 (**b**), LPAR3 (**c**), and LPAR4 (**d**) in the granulosa cells originating from healthy, transitional and atretic ovarian follicles. Small superscript letters a and b indicate significant differences in the respective mRNA ratios within the various types of follicles (*P* < 0.05) as determined by a two-way ANOVA followed by Tukey’s multiple comparison test

**Fig. 2:**
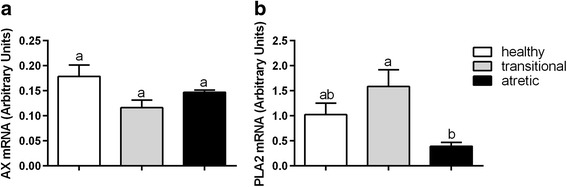
The mRNA abundance of AX (**a**) and PLA2 (**b**) in the granulosa cells originating from healthy, transitional and atretic ovarian follicles. Small superscript letters a and b indicate significant differences in the respective mRNA ratios within the various types of follicles (*P* < 0.05) as determined by a two-way ANOVA followed by Tukey’s multiple comparison test

**Fig. 3:**
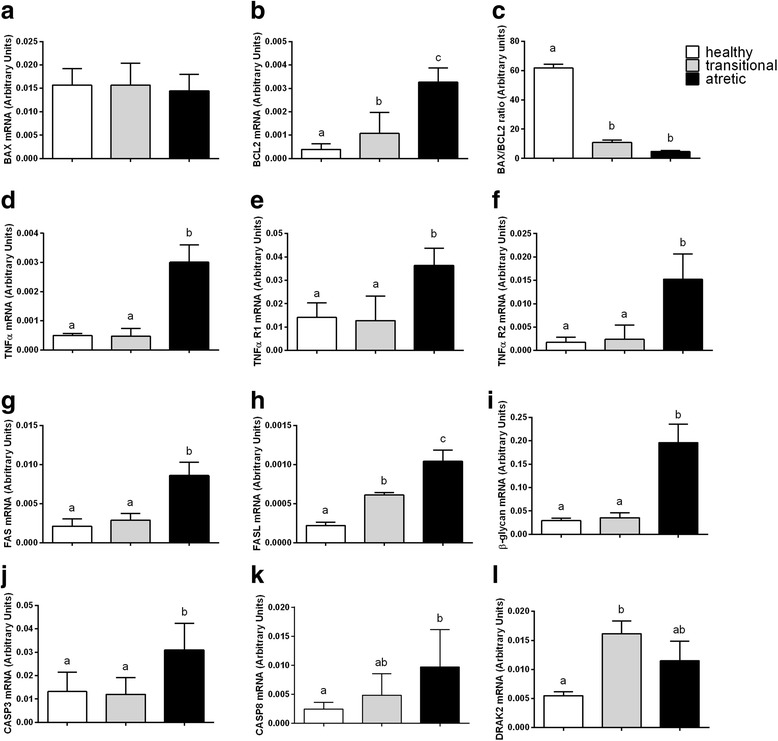
The mRNA abundance of the factors involved in apoptosis (Bax (**a**), BCL2 (**b**), Bax/BCL2 ratio (**c**), TNFα (**d**), TNFαR1 (**e**), TNFαR2 (**f**), Fas (**g**), FasL (**h**), β-glycan (**i**), CASP3 (**j**), CASP8 (**k**), DRAK2 (**l**)) in the granulosa cells originating from healthy, transitional and atretic ovarian follicles. Small superscript letters a and b indicate significant differences in the respective mRNA ratios within the various types of follicles (*P* < 0.05) as determined by a two-way ANOVA followed by Tukey’s multiple comparison test

**Fig. 4:**
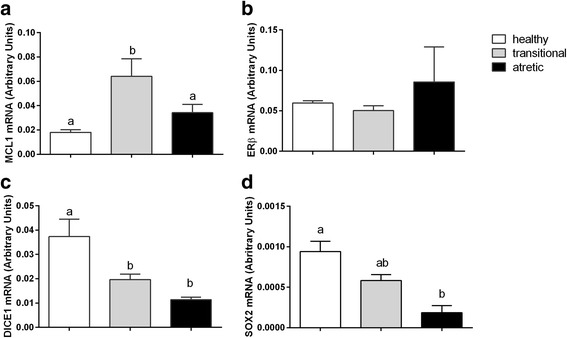
The mRNA abundance of the factors involved in cell survival processes, including MCL1 (**a**), ERβ (**b**), DICE1 (**c**), and SOX2 (**d**) in the granulosa cells originating from healthy, transitional and atretic ovarian follicles. Small superscript letters a and b indicate significant differences in the respective mRNA ratios within the various types of follicles (*P* < 0.05) as determined by a two-way ANOVA followed by Tukey’s multiple comparison test

The statistical analyses were conducted using GraphPad PRISM v. 6.0 software (GraphPad Software, Inc.). All the experimental data are shown as the mean ± SEM, and the differences were considered significantly different at the 95% confidence level (*P* < 0.05). The analyses were done using a one-way ANOVA, performed with follicle status (healthy, transitional, or atretic) as the independent classification, followed by a Kruskal-Wallis multiple comparison test (Table 2., Figs. 1, 2, 3, 4) or a correlation analysis followed by Pearson’s test (Figs. 6, 7 and 8).

**Table 2:** Distribution of ovarian follicles depending on the type (healthy, transitional, atretic)

Follicle category				
	Total	Healthy	Transitional	Atretic
Number of follicles	1028	148^a^	675^b^	205^a^
Rate of total number of follicles (%)	100	14.4^a^	65.7^b^	19.9^a^

a and b indicate significant differences in the 742 respective mRNA ratios within the various types of follicles (*P*<0.05) as determined by a two- 743 way ANOVA followed by Tukey’s multiple comparison test

## Results

### *Preliminary data:* Distribution of the ovarian follicles depending on the follicle type (healthy, transitional, atretic)

A total of 1028 bovine ovarian follicles were examined and classified into three different types, including healthy, transitional and atretic. We found that 148 follicles were defined as healthy, which was 14.4% of all the follicles, 675 follicles were defined as transitional, which was 65.7% of all the follicles and 205 were atretic follicles, which was 19.9% of the whole population of the follicles. The statistical analysis showed, that the biggest follicle population was the group of transitional follicles in comparison to the populations of healthy and atretic follicles (Table 2, *P* < 0.05).

## Lysophosphatidic acid in granulosa cells originating from different ovarian follicle types

### The expression of LPA receptors in the granulosa cells of healthy, transitional and atretic ovarian follicles

The expression levels of the mRNA of all of the types of LPA receptors examined were determined in granulosa cells of the healthy, transitional and atretic ovarian follicles (Fig. 1). The highest mRNA abundance was detected for LPAR2 (Fig. 1b, *P*<0.05). The LPAR2 and LPAR3 mRNA abundance was higher in the atretic follicles in comparison to the healthy follicles (Fig. 1a, b, *P*<0.05). The expression of LPAR4 mRNA was statistically higher in the healthy follicles in comparison to the atretic follicles (Fig. 1d, *P*<0.05). A similar abundance of LPAR1 mRNA was observed in the healthy, transitional and atretic follicle types (Fig. 1a, *P*>0.05).

### The expression of autotaxin and phospholipase A2 in the granulosa cells of the healthy, transitional and atretic ovarian follicles

The expression of mRNA of both of the enzymes involved in LPA synthesis was detected in the healthy, transitional and atretic ovarian follicles (Fig. 2a, b). A higher mRNA abundance was revealed for PLA2 in comparison to AX. The PLA2 mRNA abundance was lower in the atretic follicles compared to the healthy and transitional follicles (Fig. 2b, *P*<0.05). A mRNA abundance of AX observed in the healthy, transitional and atretic follicle types was similar (Fig. 2a, *P*>0.05).

### The immunohistochemical localization of LPARs, PLA2 and AX in the bovine ovarian follicles

**Fig. 5:**
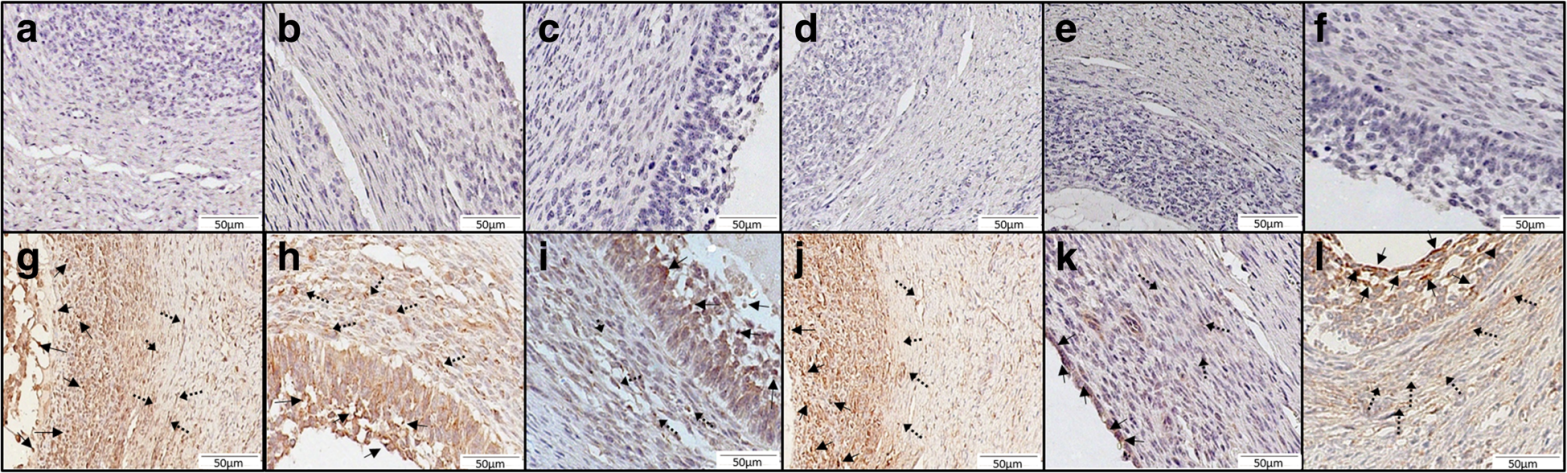
Representative micrographs of the receptors for LPA, including LPAR1 (**g**), LPAR2 (**h**), LPAR3 (**i**), and LPAR4 (**j**), and the enzymes synthesizing LPA, including AX (**k**) and PLA2 (**l**), in the antral bovine ovarian follicle. Control immunohistochemistry was performed by omitting the primary antibodies (micrographs **a**-**f**). Bars = 50 μm. The large arrows represent the granulosa cells; small arrows with a dotted line represent the granulosa cells

The immunolocation of LPAR1–4 and the enzymes involved in LPA synthesis was examined in the bovine ovarian follicles. The granulosa and theca cells from the ovarian follicles positively immunostained for all the examined LPARs (1–4) (Fig. 5). A strong signal was observed for LPAR1 (Fig. 5g), LPAR2 (Fig. 5h), LPAR4 (Fig. 5j) and PLA2 (Fig. 5l) in both granulosa and theca cells. Pale immunostaining was found for LPAR3 (Fig. 5i) and AX (Fig. 5k). No positive staining was observed in the section devoid of primary antibodies against LPARs or in the sections with immaterial antibodies (negative control: Fig. 5a-f).

## Cell survival and apoptosis factors in granulosa cells originating from different ovarian follicle types

### The expression of factors associated with apoptosis in the granulosa cells of healthy, transitional and atretic ovarian follicles

The expression levels of the mRNA of all the examined factors involved in apoptosis were detected in the healthy, transitional and atretic ovarian follicles (Fig. 3). A similar abundance of BAX mRNA was observed in the healthy, transitional and atretic follicle types (Fig. 3a, *P*>0.05). The lowest mRNA abundance for BCL (Fig. 3b, *P*<0.05) was detected in the healthy follicles, whereas the highest mRNA expression levels were observed in the follicles classified as atretic. The mRNA BAX/BCL ratio was higher in the healthy follicles in comparison to transitional and atretic types of follicles (Fig. 3c, *P*<0.05). The mRNA expression for TNFα and its receptors, TNFα R1 and TNFα R2, was statistically higher in the atretic follicle compared to the healthy and transitional follicles (Fig. 3d-f, *P*<0.05, respectively). Similar results were obtained for FAS (Fig. 3g) and FASL (Fig. 3h) mRNA expression – at higher abundance of the examined genes was found in the transitional (Fig. 3h, P<0.05) and atretic (Fig. 3g, h, *P*<0.05) follicles compared to the healthy follicles. The β-glycan mRNA abundance was seven-fold higher in the atretic follicle in comparison to healthy and transitional follicles (Fig. 3i, *P*<0.05). The highest expression levels of mRNA were also detected for CASP3 (Fig. 3j) and CASP8 (Fig. 3k) in atretic follicles compared to the healthy (Fig. 3j, k, *P*<0.05) and transitional (Fig. 3k, *P*<0.05) types of follicles. A significantly higher DRAK2 mRNA abundance was found in the transitional follicles compared to the follicles classified as healthy (Fig. 3l, *P*<0.05).

### The expression of factors associated with cell survival in the granulosa cells of healthy, transitional and atretic ovarian follicles

The mRNA expression of factors involved in cell survival and proliferation was examined in the three types of ovarian follicles (Fig. 4). The abundance of DICE1 and SOX2 mRNA was significantly higher in the healthy follicle compared to the transitional (Fig. 4a *P*<0.05) and atretic follicles (Fig. 4a, b *P*<0.05). A higher abundance of MCL1 mRNA was measured in the transitional follicle compared to the healthy and atretic follicles (Fig. 4c, *P*<0.05). The abundance of ERβ mRNA was similar among the three types of ovarian follicles (Fig. 4d *P*>0.05).

### The correlation between the mRNA expression of factors involved in proliferation/apoptosis and LPARs, PLA2 and AX in healthy, transitional and atretic ovarian follicles

**Fig. 6:**
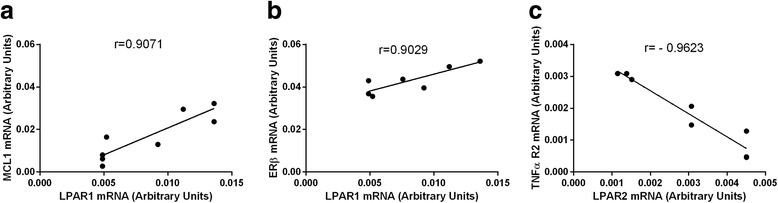
Correlations between LPARs and factors involved in the regulation of apoptosis and cell survival processes in the granulosa cells originating from the healthy ovarian follicles, including LPAR1-MCL1 (**a**), LPAR1-ERβ (**b**) and LPAR2–TNFαR2 (**c**). The correlation analysis was followed by a Pearson test

**Fig. 7:**
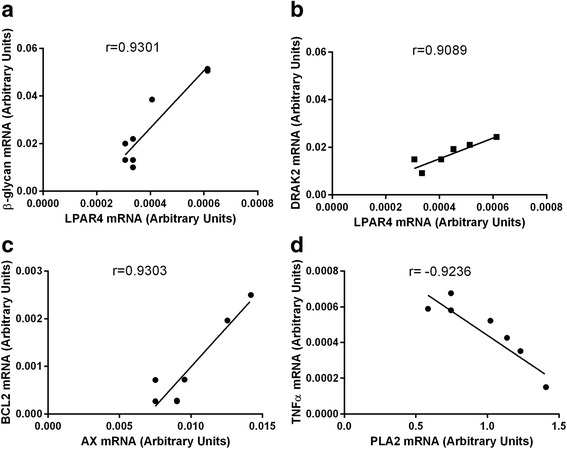
Correlations between LPARs, AX, PLA2 and factors involved in the regulation of apoptosis in the granulosa cells originating from the transitional ovarian follicles, including LPAR4-β-GLY (**a**), LPAR4- DRAK2 (**b**), AX – BCL2 (**c**) and PLA2 – TNFα (**d**). The correlation analysis was followed by a Pearson test

**Fig. 8:**
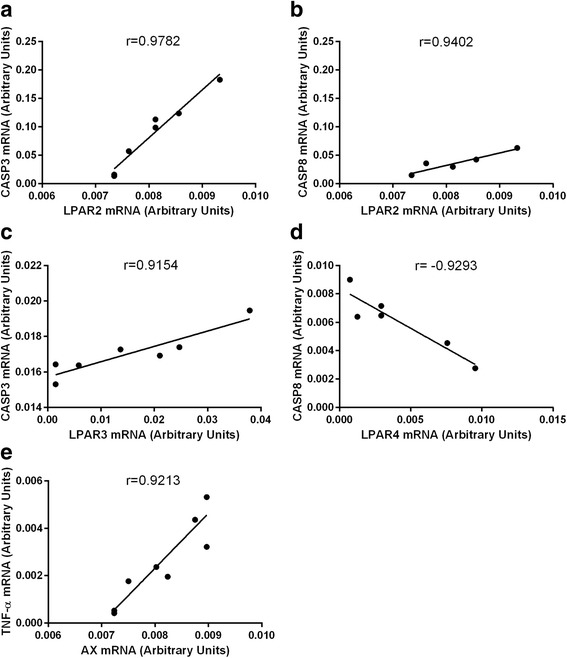
Correlations between LPARs, AX and factors involved in the regulation of apoptosis in the granulosa cells originating from the atretic ovarian follicles, including LPAR2-CASP3 (**a**), LPAR2-CASP8 (**b**), LPAR3-CASP3 (**c**), LPAR4-CASP8 (**d**), and AX-TNFα (**e**). The correlation analysis was followed by a Pearson test

The correlations between all the examined factors associated with cell survival and apoptosis, LPARs and the enzymes synthesizing LPA were investigated in the different types of ovarian follicles. In the group of healthy follicles two positive and one negative correlation between the mRNA expression of the LPARs and the factors involved in cell survival were found (Fig. 6, *P* < 0.05), respectively, as follows: LPAR1-MCL1 (Fig. 6a, r=0.9071), LPAR1-ERβ (Fig. 6b, r=0.9029) and LPAR2 –TNFα R2 (Fig. 6c, r=−0.9623). A correlations between the examined factors and the enzymes synthesizing LPA was not observed in this follicle type. Moreover, any relationship between the LPARs, PLA2 and AX and the factors associated with apoptosis was also not detected in the follicles classified as healthy. In the transitional follicles (Fig. 7, *P* < 0.05), two positive correlations between LPARs mRNA and the factors connected with cell apoptosis were investigated as follows: LPAR4-β-GLY (Fig. 7a, r=0.9301), LPAR4- DRAK2 (Fig. 7b, r=0.9089). In addition, there were two correlations between the enzymes synthesizing LPA and the factors connected with cell survival as follows: one positive between AX – BCL2 (Fig. 7c, r=0.9303) and one negative between PLA2 – TNFα (Fig. 7d, r=−0.9236). In the atretic follicles (Fig. 8, *P* < 0.05), we found the following relationships between the mRNA expression for the LPARs, AX and the factors involved in apoptosis only: LPAR2-CASP3 (Fig. 8a, r=0.9782), LPAR2-CASP8 (Fig. 8b, r=0.9402), LPAR3-CASP3 (Fig. 8c, r=0.9154), LPAR4-CASP8 (Fig. 8d, r=−0.9293) and AX-TNFα (Fig. 8e, r=0.9213). All the correlations detected in the atretic follicles were positive, except for LPAR4-CASP8 (Fig. 8d). No correlations between the LPARs, AX, PLA2 and the factors associated with cell survival were observed in ovarian follicles classified as atretic.

## Discussion

The ovarian follicle is a basic functional unit in the female reproductive physiology that is involved in the production of a mature oocyte and endocrine homeostasis. In cattle, ovarian follicles are divided into three categories, including healthy, transitional and atretic [3], but the size of the different population types was never investigated. In this study, the distribution of three different types of antral follicles was examined. We found, that the highest number of follicles (65,7%) was characteristic of the transitional group. We did not observe significant differences between the number of healthy or atretic types of follicles. However, the balance between the atretic and healthy follicles seems to be essential. Although the role of the transitional follicles is still unclear, we suggest that there might be some mechanism directing the transitional follicles to select exactly one dominant follicle, which can undergo ovulation.

There are many reports that the growth and development of follicles is regulated by endocrine hormones and locally produced growth factors. One of the local factors involved in follicle growth and development in the bovine ovary is LPA. In the cow, the expression of LPAR1–4 was confirmed in the CL [41] and in all the components of ovarian follicle, including the granulosa [15] and theca cells [42] and the cumulus-oocyte complex (COC) [43]. It is also reported that bovine granulosa cells are the source of LPA and the target of its action [15]. However, LPA expression in different follicle types was not investigated before. In present study, we confirmed the mRNA presence of LPA receptors (LPAR1–4) in the three categories of bovine ovarian follicles (healthy, transitional and atretic). The obtained results suggest that the action of LPA in the different follicle categories might be mediated via various LPARs, and the role of LPAR1 and LPAR4 seems to be leading in healthy follicles, whereas the atretic follicles are mainly under the control of LPAR2 and LPAR3. Moreover, among all the LPARs, we found that LPAR2 had the greatest mRNA expression. Similarly, in the bovine theca cells the level of mRNA for LPAR2 was significantly higher than the other LPARs, whereas the lowest mRNA expression was detected for LPAR3 [42]. Boruszewska et al. [43] also described the greatest mRNA level for LPAR2 in the COCs isolated from the bovine ovarian follicles, which may indicate, that LPAR2 plays the most significant role in LPAR-mediated signaling in the bovine ovarian follicle. Furthermore, we confirmed the immunolocalization of LPAR1–4, both in the granulosa and theca cells of the antral ovarian follicles. The results of our experiment indicated that the granulosa cells, in addition to the theca and COCs, represent targets for LPA action in the different types of bovine ovarian follicles.

The immunolocalization and mRNA expression of the enzymes involved in LPA production – AX and PLA2 – was also confirmed in the granulosa cells in all the examined types of follicles. A significantly greater PLA2 mRNA expression, in comparison to AX mRNA in the healthy, transitional and atretic follicles was detected, which suggests that PLA2 plays a major role in LPA production in the granulosa cells of the bovine ovarian follicle. Moreover, we noticed significant differences between the examined groups of follicles in PLA2 expression, but not in AX expression. A greater mRNA abundance was documented in the healthy and transitional follicles in comparison to the atretic follicles, indicating a greater possibility of LPA synthesis in the follicles predestined to be dominant or in the follicles not determined yet compared to the atretic follicles. The principal involvement of PLA2 in LPA synthesis in the granulosa cells is consistent with our recent results in the bovine theca cells, in which the PLA2 expression, in comparison to AX, was significantly greater in all the examined follicle types [42].

The basis of the dynamic changes during follicular development is apoptosis and processes involved in the cell survival. Ovarian follicular atresia is initiated with the apoptosis of granulosa cells [9]. Apoptosis proceeds through two main pathways, including the activation of CASP8, initiating the cascade of effector caspases, and the mitochondrial pathway, which is controlled by the regulatory proteins BAX and BCL2, which belong to the BCL2 family [44]. In the present study, we found no differences in the expression of BAX in the different types of follicles, but, surprisingly, we detected a significantly higher expression of antiapoptotic BCL2 in the atretic follicle in comparison to the healthy follicles. Moreover, we found a higher BAX/BCL2 ratio in the healthy follicles compared to the atretic follicles. Taking into consideration, that after follicle selection, only one dominant follicle continues to grow and development [45], whereas more than 99% of the follicles, including the major part of the healthy follicles, undergo atresia [4, 46], we suggest the presence of some mechanism controlling the balance between different types of follicles, based on the regulation of BCL-family gene expression. In our study, we demonstrated a strong positive correlation between the expression of BCL2 mRNA and AX mRNA, which was observed in the transitional type of the bovine ovarian follicles. Even though the transitional follicles comprised the biggest group of the bovine ovarian follicles, their role in ovarian physiology is still not thoroughly understood. We postulated that the plurality of the developing follicles in each reproductive cycle, with the possibility of ovulation of only one of them, suggests that the transitional follicles are the temporary stadium, aiming towards the atretic follicle type. Therefore, the correlation between antiapoptotic BCL2 and AX, the enzyme synthesizing LPA in the transitional follicles may suggest that, during the process of follicle determination, the augmented LPA synthesis in the follicles from the transitional group might also contribute to redirecting at least some of the follicles towards the healthy group of follicles.

Apoptosis, on the receptor level, can be initiated via several cytokines and their receptors, including tumor necrosis factor- alpha (TNFα) and TNF receptors (TNFR). Interestingly, TNFα induces both cell apoptosis and proliferation by binding to receptors 1 or 2, respectively [47]. The presence of TNFα was already reported in the oocyte and the granulosa and theca cells [48–50]. The present study exhibited the mRNA levels of TNFα, TNFαR1 and TNFαR2 in the granulosa cells isolated from the different types of bovine ovarian follicles. The increased mRNA level of TNFα and the TNFRs in the granulosa cells acquired from the atretic follicles was examined in comparison to the transitional and healthy follicles. While the TNFα and TNFαR1 mRNA expression results agreed with the expected role of these molecules in the healthy and atretic follicles, the data on the mRNA abundance of TNFαR2 was surprising. We presume that the same expression pattern of TNFαR2, mediating mainly antiapoptotic effects, and proapoptotic TNFαR1 in the granulosa cells isolated from atretic follicles might result from the implication of these two receptors in apoptosis in the atretic follicles. However, the level of TNFαR1 expression was two times higher than TNFαR2 expression, which accounts for the dominant role of TNFαR1 in apoptosis in the bovine atretic ovarian follicles. Moreover, in the granulosa cells of the healthy follicles, a negative relationship between TNFα and LPAR2 (*r* = −0.9623) was detected. We also revealed a negative correlation between TNFα and PLA2 (*r* = −0.9636) in the granulosa cells of the transitional follicles, whereas the positive association between TNFα and AX was confirmed in the atretic follicles (*r* = 0.9213). On the other hand, Evans et al. [50] demonstrated previously that the mRNA expression of TNFα was elevated in the bovine granulosa cells of the subordinate in comparison to the dominant follicles, which implies that TNFα induced follicular atresia in the ovary. These data suggest that the assisting role of LPA towards the TNFα-dependent regulation of apoptosis in the granulosa cells of the atretic and transitional follicles rather than in the healthy ovarian follicles.

In our study, we also confirmed that the follicle type depended on the mRNA expression of another transmembrane receptor, which mediates apoptosis, namely FAS and its ligand in the bovine ovary. The highest level of FAS and FASL mRNA was documented in the atretic follicle, whereas the lowest expression was in the healthy follicle types. These results are consistent with the data by Porter et al. [51], who documented significantly higher FAS antigen mRNA levels in the granulosa cells from the subordinate follicle in comparison to the dominant follicles, suggesting a fundamental difference in granulosa cell susceptibility to FAS antigen-mediated death. Our results are also in agreement with the studies by Kim et al. [52] and Kondo et al. [53] showing an elevated expression of FAS antigen in the granulosa cells of the atretic follicles in comparison with the healthy follicles in rats and humans. Moreover, granulosa cells can also promote follicular development by downregulating death-induced FAS/FASL expression [54], implying that the FAS system is one of the most important apoptosis regulators in the ovarian follicle. The follicle type dependent mRNA expression was also confirmed for DRAK2, a serine/threonine kinase, that has homologous domains with TNFα and the FAS antigen.

The essential role of CASP3 and CASP8 in the mitochondrial pathway of apoptosis is well described in the literature. Follicular atresia, in a low FSH environment, involves the induction of CASP3 expression and cleavage, leading to granulosa cell death [55]. High levels of CASP8 directly initiate the cleavage of an effector caspase, CASP3, thereby initiating the execution phase of apoptosis [44]. In the present study, we demonstrated that the inherence of the follicle-type depended on CASP3 and CASP8 mRNA in the bovine granulosa cells. We found a significantly higher expression of both of the examined caspases in the granulosa of the atretic follicles in comparison to the transitional and healthy follicles. Furthermore, we observed at number of correlations between CASP3, CASP8 and LPARs mRNA transcript levels in the atretic ovarian follicles. We detected positive dependences between CASP8 and LPAR2 (*r* = 0.9402), CASP3 and LPAR2 (*r* = 0.9782) as well as CASP3 and LPAR3 (*r* = 0.9154). A negative correlation between CASP8 and LPAR4 (*r* = −0.9293) was also found in the granulosa cells of the atretic follicles. The above relationships document the participation of LPA in the regulation of caspase-induced apoptosis in the atretic type of ovarian follicles via enhancing its activity by LPAR2 and LPAR3 or via silencing by LPAR4.

β-glycan (the type III TGF receptor, TGFBR3) is a membrane-bound proteoglycan, which serves as an additional TGF superfamily receptor [56]. The mRNA expression of β-glycan was follicle-type dependent, and its mRNA transcript abundance was statistically higher in the atretic follicles in comparison to the healthy and transitional follicles. These results agree with the data [50, 57], documenting a greater mRNA level of β -glycan in the theca and granulosa cells in the subordinate follicles in comparison to the dominant follicles and suggests that, in the subordinate follicles, β-glycan can be the mediator of inhibin activity to extend androgen production by the theca cells and suppress estradiol production by the granulosa cells. In the present study, we demonstrated a positive correlation of β-glycan and LPAR4 in the granulosa cells of the transitional ovarian follicles (*r* = 0,9301). The coexpression of LPAR4 and β -glycan suggests that LPA is involved in the modulation of estradiol production by the granulosa cells. This correlation, in the transitional follicles, might imply that the ability of inhibins, acting via β–glycan, together with LPA, acting via LPAR4, shifts the balance between the number of healthy and atretic follicles.

In summary, the deprivation of key survival factors or the stimulation by death ligands is the main cause of apoptosis in the bovine ovarian follicle [58]. On the other hand, it was previously well documented that the apoptosis of the granulosa cells initiates follicular atresia, which is triggered by the action of the death ligand receptor systems, such as FASL, FAS antigen, TNFα, its receptors and BCL2 family proteins [9, 58, 59]. Subsequently, these signals induce different intracellular pro-apoptotic pathways, which result in the cleavage of CASP3, leading to apoptosis [9]. In our study, we documented, for the first time, the expression of the above-mentioned factors in the granulosa cells isolated from three functionally different types of ovarian follicles. Moreover, we confirmed the follicle type-dependent expression of the pro-apoptotic factors, which suggests various intensities of apoptosis during folliculogenesis depending on the role and destiny of the follicle. We were also the first to document the relationship between LPA synthesis and the action and expression of genes involved in apoptosis in the granulosa cells in three different types of ovarian follicles.

In addition to cell death, the dynamic changes in follicular development are caused by the action of factors involved in cell survival and proliferation. In this study, we examined the expression of DICE1, MCL1, SOX2 and ERβ, in the granulosa cells of the three types of bovine ovarian follicles.

DICE1 is the RNase III cytoplasmatic enzyme required for the processing of small regulatory RNAs [58, 60]. Although, the function of DICE1 and its products is widely studied in the female reproductive tract [61, 62], its role in the ovarian follicle is still poorly understood. The present study showed the expression of DICE1 in the bovine granulosa cells originated from healthy, transitional and atretic types of follicles. We found a significantly higher DICE1 mRNA expression in the granulosa cells of the healthy follicles in comparison to transitional and atretic follicles. Our results agree with Evans et al. [50] who documented a higher DICE1 mRNA expression in the dominant follicles in comparison to the subordinate follicles. Other authors also documented that the loss of DICE1 expression in ovarian granulosa cells reduced the ovulation rates, induced follicle degeneration and elevated the number of atretic follicles in mice [59, 63, 64]. In our study, we did not find any relationship between DICE1 mRNA expression and LPA synthesis and action in the granulosa cells of the various types of ovarian follicles.

Similar results were documented for the sex-determining region Y-box 2 (SOX2), which belongs to the family of transcription factors involved in cell proliferation, pluripotency and differentiation [65]. We found the highest expression of SOX2 mRNA in the granulosa cells originated from the healthy follicles, whereas the lowest transcript level of SOX2 was observed in the granulosa cells of the atretic follicles. These data indicate the significance of granulosa cell proliferation in healthy follicles and the role of DICE1 and SOX2 in this process.

The anti-apoptotic BCL2 family proteins are pivotal regulators of the mitochondrial apoptotic pathway. Recent studies highlight the critical role of the BCL2 family member called myeloid cell leukemia −1 (MCL1) [66, 67]. MCL1 expression was identified in the ovaries of humans [68], rodents [66, 69] and ruminants [50, 70]. In our study, we documented a higher mRNA transcription of MCL1 mRNA in the transitional follicles in comparison to the healthy and atretic types of follicles. The obtained results are in opposition to the results of Evans et al. [50] who demonstrated greater MCL1 mRNA expression in the granulosa cells of the dominant follicles in comparison to the subordinate follicles. However, in the healthy follicle category, only one of the follicles is dominant, which can be the reason for this result discrepancy. Although the expression of antiapoptotic MCL1 mRNA was not higher in the healthy follicle category, we found a positive correlation between MCL1 and LPAR1 mRNA expressions (*r* = 0.9071) in the granulosa cells in the healthy follicle category. These data indicate that, in the healthy follicles, LPA, acting via LPAR1, might regulate MCL1 expression leading to the stimulation of the anti-apoptotic processes.

It was well documented previously that estradiol playes a key role in the selection and sustained growth of the dominant follicle [71, 72], which is reflected by the expression of the estradiol receptors in the granulosa and theca cells [73]. Among the two estradiol receptors (ERα and ERβ) described in the literature, ERβ is the dominant ovarian subtype. In the present study, the expression of ERβ mRNA in the different bovine ovarian follicle types was confirmed. Although, we did not detect any statistically significant differences in the ERβ mRNA levels in the granulosa cells of the three types of follicles, we revealed a strong association between ERβ and LPAR1 mRNA levels (*r* = 0.9029) in the granulosa cells of the healthy follicles. This correlation indicates that, in the healthy follicles, LPA, acting via LPAR1, might interact with estradiol, stimulating ERβ mRNA expression leading to the stimulation of the granulosa cell differentiation and proliferation. These data agree with Boruszewska et al. [15] who also documented a positive LPA influence on estradiol production in bovine granulosa cells.

## Conclusions

To summarize, in our study we documented the follicle type-dependent expression of the receptors for LPA and the enzymes involved in LPA production in bovine granulosa cells. Moreover, we investigated the relationship between LPA synthesis and the action and expression of the genes involved in cell apoptosis and survival in the granulosa cells in three different types of ovarian follicles.

With regard to follicle atresia, we found that LPA assists in theTNFα-dependent regulation of apoptosis in the granulosa cells of the atretic and transitional ovarian follicles. Moreover, we found that LPA participate in the regulation of caspase-induced apoptosis in the atretic type of ovarian follicles via enhancing its activity by LPAR2 and LPAR3 or via silencing by LPAR4. With regard to cell survival in the different functional types of ovarian follicles, LPA, acting via LPAR1, might regulate MCL1 expression, leading to the stimulation of the anti-apoptotic processes in the healthy follicle type. In addition, in the healthy follicles, LPA, acting via LPAR1, might interact with estradiol to stimulate ERβ mRNA expression, leading to the stimulation of granulosa cell differentiation and proliferation.

## Additional file

Additional file 1: The supplementary information citation. (PDF 655 kb)

## Abbreviations

AX: Autotaxin
BAX: BCL2 associated X, apoptosis regulator
BCL2: B-cell lymphoma 2, apoptosis regulator
CASP3: Caspase 3
CASP8: Caspase 8
CL: Corpus luteum
COC: The cumulus-oocyte complex
DICE: Integrator complex subunit 6
DRAK2: Serine/threonine kinase 17b
E2: Estradiol
ERβ: Estradiol receptor β
FAS: Cell surface death receptor
FASL: FAS ligand
FF: Follicular fluid
FGF: Fibroblast growth factor
FSH: Follicle stimulating hormone
FSHR: FSH receptor
IGF: Insulin-like growth factor
LH: Luteinizing hormone
LPA: Lysophosphatidic acid
LPAR: LPA receptor
MCL: Sex-determining region Y-box 2
P4: Progesterone
PLA: Phospholipase A
SOX: Sex-determining region Y-box 2
TGF: Transforming growth factor
TNFa: Tumor necrosis factor- alpha
TNFaR1: Tumor necrosis factor- alpha receptor 1
TNFaR2: Tumor necrosis factor- alpha receptor 2
