# Correction to: Expression of factors involved in apoptosis and cell survival is correlated with enzymes synthesizing lysophosphatidic acid and its receptors in granulosa cells originating from different types of bovine ovarian follicles

**DOI:** 10.1186/s12958-017-0298-6

**Published:** 2017-10-09

**Authors:** Emilia Sinderewicz, Katarzyna Grycmacher, Dorota Boruszewska, Ilona Kowalczyk-Zięba, Joanna Staszkiewicz, Tomasz Ślężak, Izabela Woclawek-Potocka

**Affiliations:** Department of Gamete and Embryo Biology, Institute of Animal Reproduction and Food Research, Polish Academy of Sciences, 10-747 Olsztyn, Poland

## Correction

After publication of the original article [1] it was noted that the caption of Table two was incorrect. This caption should read “Distribution of ovarian follicles depending on the type (healthy, transitional, and atretic). Small superscript letters a and b indicate significant differences in the respective ovarian follicle numbers within the various types of follicles (*P* < 0.05) as determined by a one-way ANOVA followed by the Kruskall-Wallis test.” Table 2 and the correct caption are shown below:

**Table 2:** Distribution of ovarian follicles depending on the type (healthy, transitional, atretic)

		Follicle category		
	Total	Healthy	Transitional	Atretic
Number of follicles	1028	148^a^	675^b^	205^a^
Rate of total number of follicles (%)	100	14.4^a^	65.7^b^	19.9^a^

Small superscript letters a and b indicate significant differences in the respective ovarian follicle numbers within the various types of follicles (*P* < 0.05) as determined by a one-way ANOVA followed by the Kruskall-Wallis test

These errors were introduced during typesetting, thus the publisher apologizes for this error.
