# Supplementary material for: Expression of factors involved in apoptosis and cell survival is correlated with enzymes synthesizing lysophosphatidic acid and its receptors in granulosa cells originating from different types of bovine ovarian follicles
Source: Reprod Biol Endocrinol. 2017 Sep 6;15:72. doi: 10.1186/s12958-017-0287-9 (PMC5586021; doi:10.1186/s12958-017-0287-9)
Supplement: Additional file 1: — The supplementary information citation. (PDF 655 kb) [file 12958_2017_287_MOESM1_ESM.pdf]

AMERICAN JOURNAL EXPERTS

PAID ON 06/26/2017 8:34 AM

American Journal Experts

Send to:
Emilia Sinderewicz
Institute of Animal Reproduction and Food Research
Tuwima 10 Str.
10-748 Olsztyn
Olsztyn, warminsko-mazurskie
Poland, 10-748

American Journal Experts
601 West Main Street, Suite 102
Durham, NC 27701, United States
Phone: 1-919-704-4253
Fax: 1-919-287-2439
http://www.aje.com
Email: support@aje.com
Tax ID: 412141424

Invoice

Receipt code: **KAPRE-6F8-0626083351**
Authors: Emilia Sinderewicz, Katarzyna Grycmacher, Dorota Boruszewska, Ilona Kowalczyk-Zięba, Joanna Staszkiewicz, Tomasz Ślężak, Izabela Woclawek-Potocka
Title: Expression of factors involved in apoptosis and cell survival is correlated with enzymes synthetizing lysophosphatidic acid and its receptors in granulosa cells originated from different types of bovine ovarian follicles
Submission date: June 26 2017, 08:33 am

Invoice date	Description	Length	Time	Area of study	Price
June 26, 2017	Standard Editing	Standard (3501 - 6000 words)	5 days	Cell Survival and Cell Death	$268.00
				Total credit card charges	$268.00
				Remaining balance	$0.00

PAYMENT METHOD(S): Credit Card
TERMS: Net 30 days. Online order
NOTES:
This invoice has already been paid, and is for your internal records only. Thank you for choosing American Journal Experts.
